# Multi-Objective Optimization of Curing Profiles for CFRP Patch Repair Under Thermochemical Coupling

**DOI:** 10.3390/polym18182188

**Published:** 2026-09-08

**Authors:** Ning Han, Yuan Wang, Yungang Sun, Erliang Liu, Longxin Fan

**Affiliations:** 1 Technology Center, State-Owned Wuhu Machinery Factory, Wuhu 241000, China; 2 School of Mechanical Engineering, Changzhou University, Changzhou 213164, China

**Keywords:** CFRP, curing process, sensitivity analysis, multi-objective optimization, finite element simulation

## Abstract

This study addresses the challenge of temperature non-uniformity during carbon fibre-reinforced polymer (CFRP) composite patch repair, which compromises curing quality and process efficiency. A coupled heat transfer–curing kinetics finite element model was developed and experimentally validated to investigate the heat sink effect of support structures. Key findings reveal that temperature differences concentrate near aluminum components and increase with curing temperature. For the present scarf-repair configuration, global sensitivity analysis identified the second-stage holding temperature (*T*_2_) and heating rate (*r*_2_) as the dominant factors governing temperature uniformity, whereas the holding times (*dt*_1_ and *dt*_2_) primarily determine the total curing time (*t*_total_). A novel multi-objective optimization framework combining optimal Latin hypercube sampling, radial basis functions, and NSGA-II was established. The optimized curing profile achieves a surrogate-predicted reduction of 22.5% in maximum temperature difference (22.0% when confirmed by high-fidelity finite element verification) and 36% in total curing time, while maintaining a minimum degree of cure above 0.98. These results provide a validated, surrogate-based framework for designing curing protocols that resolve metal-induced thermal non-uniformity in composite repairs without sacrificing cure quality.

## 1. Introduction

Carbon fiber-reinforced polymers (CFRPs) are extensively utilized in modern aircraft primary structures, including wings and fuselages, owing to their superior specific strength, fatigue resistance, and design flexibility [1,2,3,4]. During prolonged service, these composite components are susceptible to damage from impacts, lightning strikes, and cyclic loading. Scarf repair, which restores both aerodynamic contour and load-bearing capacity, has emerged as the preferred technique for structural restoration, typically employing vacuum bag curing to achieve an integrated load path between the patch and parent structure [5,6,7,8].

The integrity of scarf repairs critically depends on precise temperature control during the curing cycle, which drives the cross-linking reaction of the patch–adhesive interface [9,10]. However, aircraft repair configurations present a fundamental challenge: the repair zone inherently comprises a multi-material interface, bonding CFRP patches to underlying metal substructures (e.g., aluminum ribs) [11]. The high thermal diffusivity of aluminum alloys (Al) induces a pronounced “heat sink effect,” locally accelerating heat dissipation and reducing heating rates near the bottom surface of the patch at the patch–adhesive interface [12]. This thermal heterogeneity generates significant temperature gradients across the interface, potentially leading to localized undercuring or elevated residual stresses—both detrimental to long-term structural reliability [13,14]. This heat sink effect is further amplified by the pronounced anisotropy of CFRP thermal conductivity: the in-plane conductivity (k_xx_) of the composite is nearly an order of magnitude higher than its through-thickness conductivity (k_yy_, k_zz_), whereas aluminum alloys conduct heat isotropically and roughly two orders of magnitude faster than the composite through-thickness direction. This conductivity mismatch means heat is preferentially channeled away from the patch–adhesive interface along the metallic substructure while the composite itself resists through-thickness heat flow, compounding the interfacial temperature nonuniformity beyond what an isotropic-material analysis would predict.

While previous studies have established that heating rate and curing temperature critically influence residual stress distributions in repaired structures [15,16], a trade-off persists between process efficiency and interface quality. Numerical simulation combined with multi-objective optimization has proven effective for controlling composite curing processes. Recent advances have enabled simultaneous optimization of curing time, temperature uniformity, and degree of cure (DoC) for thick-section composites [17,18], complex-shaped components [19,20], and specialized repair scenarios [21,22]. For instance, surrogate-assisted optimization frameworks employing neural networks have demonstrated significant reductions in process time and residual stresses for laminated structures [17,23]. Similarly, non-dominated sorting differential evolution algorithms have been developed to minimize thermal gradients and cure time for aircraft components [19]. Machine learning-enhanced surrogate modeling has achieved exceptional prediction accuracy (R^2^ > 0.998) for curing processes, enabling millisecond-level predictions with over 10^4^× speed-up compared to finite element simulations [24]. Physics-informed neural networks have also been successfully applied to optimize out-of-autoclave curing processes while adhering to production constraints [25]. Nevertheless, existing research predominantly addresses primary composite manufacturing or generic repair scenarios. The unique multi-physics challenge of patch repairs on operational aircraft—specifically, the coupled heat transfer–curing kinetics at metal–composite interfaces and the sensitivity of patch–adhesive interface thermal uniformity to structural heat sink effects—remains inadequately characterized [26,27]. Systematic elucidation of these interactions is essential for developing robust, high-quality repair protocols for aerospace composite structures [28].

To close this gap, the present study develops a comprehensive framework for investigating and optimizing the thermal-curing behavior of CFRP scarf repairs. The bottom surface of the patch, which is directly in contact with the adhesive film, is referred to as the patch–adhesive interface in this study. An experimentally validated, coupled heat transfer–curing kinetics finite element model quantifies the influence of metallic substructures on the evolution of temperature at the patch–adhesive interface. Global sensitivity analysis identifies the dominant parameters governing temperature uniformity, curing time, and DoC at the patch–adhesive interface. A multi-objective optimization strategy—integrating optimal Latin hypercube sampling, RBF surrogate modeling, and NSGA-II—then minimizes both maximum temperature difference (Δ*T*) and total curing time (*t*_total_) while ensuring full cure (DoC > 0.98). While the specific dominant parameters and quantitative improvements reported here are established for the tested scarf geometry, aluminum-rib support structure, and material system, the underlying framework—and the expectation that peak-temperature and heating-rate parameters govern heat-sink-driven interfacial gradients in any configuration pairing a high-conductivity metal with a low-through-thickness-conductivity composite—is expected to generalize. This study provides a systematic investigation of metal-induced thermal heterogeneity in composite patch repairs.

## 2. Finite Element Model

### 2.1. Description of Heat Transfer Process

During composite patch repair, thermal input originates from external electric heating blankets and internal exothermic resin crosslinking. The curing process therefore involves heat conduction coupled with a nonlinear internal heat source—specifically, the reaction enthalpy of the matrix resin. Accordingly, the finite element model integrates heat transfer and curing kinetics modules, with the degree of cure (DoC) and temperature field evaluated via a thermochemically coupled heat transfer analysis.

(1) $$\rho_{c}C_{c}\frac{\partial T}{\partial t}=k_{xx}\left(\frac{\partial^{2}T}{\partial x^{2}}\right)+k_{\mathrm{yy}}\left(\frac{\partial^{2}T}{\partial y^{2}}\right)+k_{zz}\left(\frac{\partial^{2}T}{\partial z^{2}}\right)+\rho_{r}(1-V_{f})H_{R}\frac{d\alpha }{dt}$$

where $\rho_{c}$, $C_{c}$, and $k_{ii}(i=x,\;y,\;z)$ represent the density, specific heat capacity, and thermal conductivity of the composite material, respectively; $V_{f}$ denotes the fiber volume fraction in the composite; $\rho_{r}$ is the density of the resin; $H_{R}$ is the total heat released per unit mass of resin during the curing reaction; *t* and *T* denote the current temperature and curing reaction time, respectively. $\frac{d\alpha }{dt}$ represents the instantaneous DoC, which can be calculated using the resin’s curing kinetics model during the incremental steps of the finite element analysis.

Curing kinetic models are broadly classified into phenomenological and mechanistic categories. Phenomenological models describe the overall reaction using a simplified single-reaction representation, whereas mechanistic models emphasize detailed analysis of the underlying kinetic mechanisms. Due to its simplicity and effectiveness, the phenomenological approach is widely adopted to simulate resin curing behavior. The Kamal–Sourour autocatalytic model, a representative phenomenological formulation, is employed in this study, with its kinetic expression given in [23,24].

(2) $$\frac{d\alpha }{dt}=(k_{1}+k_{2}\alpha^{m})\cdot {\left(1-\alpha \right)}^{n}$$

The model describes the shape of the heat flow curve (related to the type of reacting resin) and the temperature-dependent Arrhenius-type function K(T), with the relationship expressed as follows [23]:

(3) $$k_{i}(T)=A_{i}\cdot exp(\frac{-E_{i}}{TR})$$

In the above equation, *A*_i_ denotes the prefactor, $E_{i}$ represents the apparent activation energy of the curing reaction, *R* is the universal gas constant, and *T* is the temperature.

### 2.2. Finite Element Simulation

**Figure 1:**
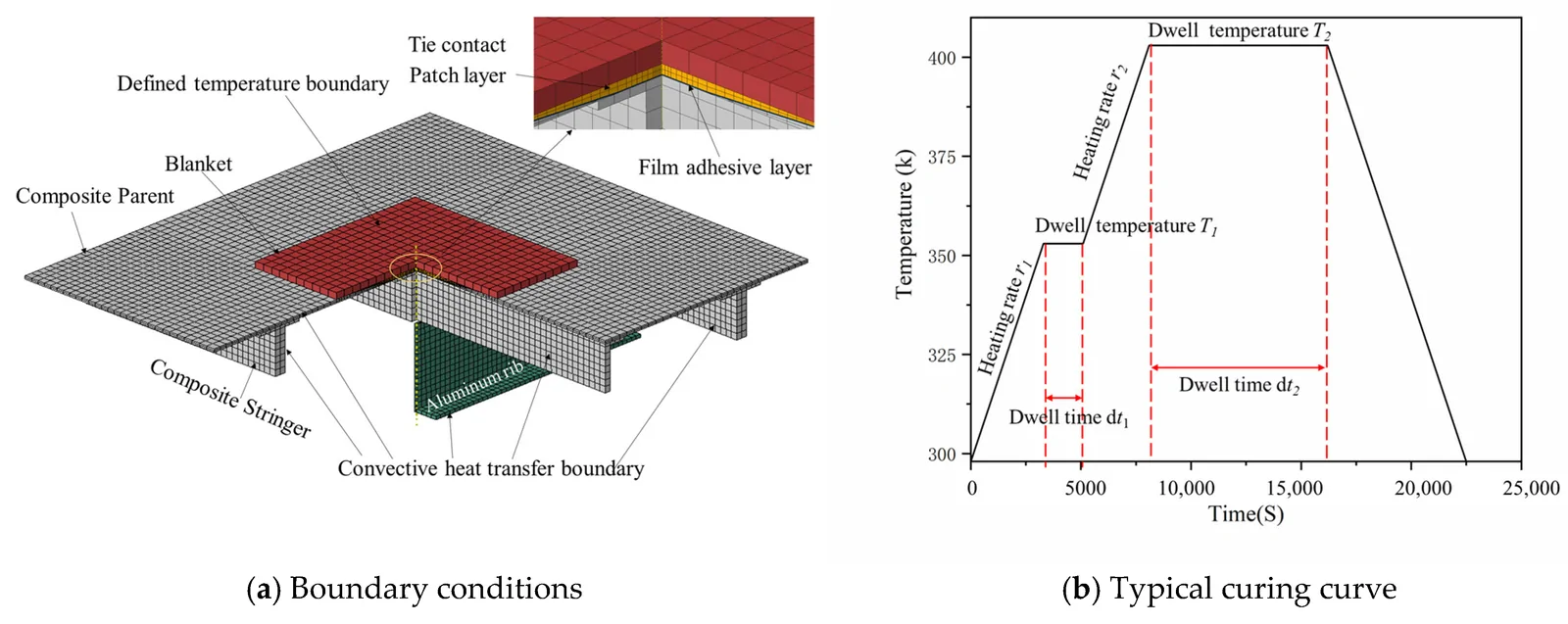
Establishment of the finite element model.

The curing process was simulated using ABAQUS 2017 in conjunction with FORTRAN user subroutines. Eight-node linear brick elements (DC3D8) were employed throughout the model. The patch was assigned a 2.5 mm target element size, with the minimum element size constrained to 0.25 mm (one-tenth of the target size) to allow automatic refinement in regions of high geometric curvature. All remaining components of the assembly were assigned a coarser 5 mm target element size, resulting in a total of 18,814 elements (Figure 1a). A mesh sensitivity analysis was performed by refining the target element size to 1.5 mm and 1 mm; the resulting temperature fields showed no significant change relative to the 2.5 mm mesh, confirming that the adopted mesh density yields stable, mesh-independent results while retaining computational efficiency.

Four subroutines were employed: HETVAL to define the internal heat generation from the curing reaction; USDFLD to model the evolution of the degree of cure; DISP to apply time-dependent temperature boundary conditions; and FILM to prescribe convective heat transfer conditions on exposed surfaces. Heat flux continuity across all material interfaces was enforced using tie constraints in ABAQUS. The coupled thermal-cure equations were solved using ABAQUS’s implicit Newton-Raphson with automatic time incrementation, subject to a maximum temperature change of 2 °C per increment and a nonlinear convergence tolerance of 5 × 10^−3^ on the residual heat flux, ensuring numerical stability throughout the strongly exothermic curing stages [29]. The ambient temperature was set to 25 °C, and a convective heat transfer coefficient of *h* = 10 W/(m^2^·K) was applied at all exposed surfaces to represent natural convective heat exchange with the surrounding environment [30].

The temperature boundary conditions were set based on the curing process curves of the patch and adhesive film, comprising the following five stages (Figure 1b): (a) an initial temperature of 25 °C, heated to 80 °C at a heating rate of 1.0 °C/min (*r*_1_); (b) a hold at 80 °C for 30 min (*T*_1_, *dt*_1_); (c) heating to 130 °C at a rate of 1.0 °C/min (*r*_2_) (*T*_2_); (d) a hold at 130 °C for 135 min (*T*_2_, *dt*_2_); and (e) cooling to 25 °C at a rate of −1.0 °C/min.

### 2.3. Experimental Validation

**Figure 2:**
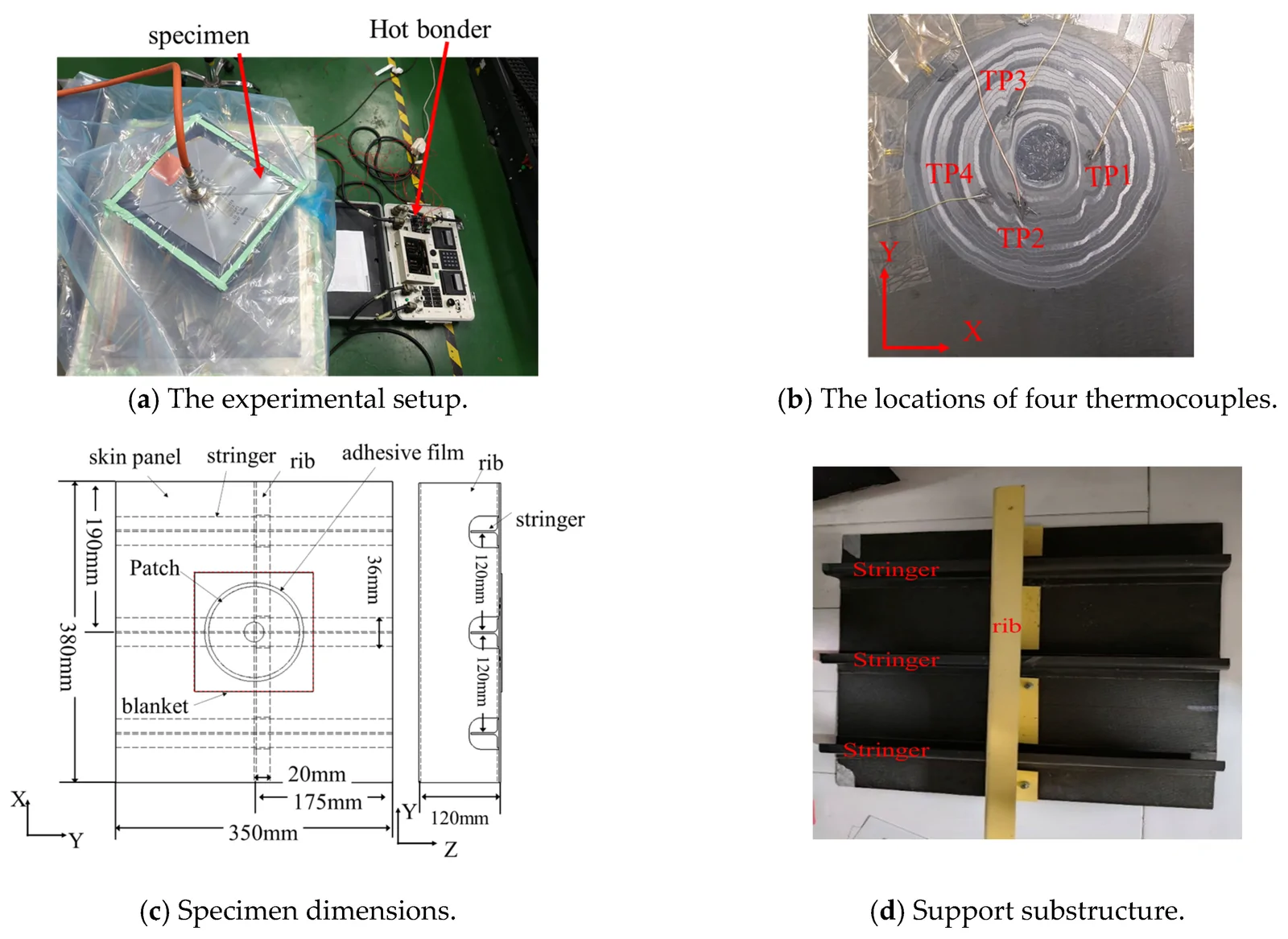
Test specimen and instrumentation.

The experimental setup employed an HCS9200B hot bonder (Heatcon Composites, Tukwila, WA, USA) as the heating source. Bondline temperatures were recorded using an NI cDAQ-9171 USB data acquisition system equipped with an NI 9211 thermocouple input module, as illustrated in Figure 2a. Type K thermocouples, with a manufacturer-specified accuracy of ±0.5 °C, were embedded within the adhesive bondline to monitor the curing temperature history, and their locations are shown in Figure 2b. To verify the repeatability of the measurements, three independently fabricated specimens were tested under identical experimental conditions.

The repair assembly consisted of a skin panel, one aluminum rib, three composite stringers, an adhesive film, and a composite patch, as illustrated in Figure 2c. The overall dimensions of the assembly were 380 mm × 350 mm × 120 mm. A scarf repair with a top diameter of 25 mm and a base diameter of 125 mm was introduced in the damaged region. The rear surface of the skin panel was reinforced by a support substructure comprising three composite stringers and one aluminum rib, as shown in Figure 2d, thereby reproducing the local stiffened configuration typically found in aerospace composite structures. During the repair process, a 150 mm × 150 mm electric heating blanket was positioned directly above the repair region (Figure 2c). The rib was machined from 7075 aluminum alloy, and LWF-2B epoxy adhesive film was used to bond the repair patch to the parent laminate. The thermophysical properties of the adhesive film and aluminum alloy are summarized in Table 1.

In the thermal model, the skin panel represented the original parent structure, which had been fully cured prior to repair. Consequently, it was assumed to be chemically inert throughout the repair curing cycle and contributed only its thermophysical properties to the heat transfer analysis. Cure kinetics were therefore assigned only to the ACTECH 1206 patch resin. The material properties adopted in the numerical model are listed in Table 2.

The 0.2 mm thick LWF-2B adhesive film was modeled using only its thermophysical properties, without explicitly considering cure kinetics or the associated exothermic heat generation. Because of its small thickness relative to the surrounding laminates, its characteristic thermal diffusion time (~0.1 s) is several orders of magnitude shorter than the duration of the curing dwell stages, so any heat released during adhesive curing rapidly equilibrates within the adjacent laminates. This is confirmed quantitatively by an order-of-magnitude estimate using the classical thin-slab solution for uniform internal heat generation, Δ*T*_max_ ≈ q‴L^2^/(8k) [18]; the resulting through-thickness temperature rise from adhesive self-heating is on the order of 10^−3^ °C—roughly four orders of magnitude below the ΔT values of interest (15.5–20 °C), which arise from the macroscale heat-sink effect of the metallic rib over centimeter length scales and minute-to-hour time scales. The simplification therefore has a negligible influence on the reported patch–adhesive interface temperature field.

## 3. Parameter Sensitivity Analysis and Multi-Objective Optimization

### 3.1. Parameter Sensitivity Analysis

To identify the dominant parameters governing temperature non-uniformity at the patch–adhesive interface during composite patch repair and to quantify both individual and interactive effects, a global sensitivity analysis framework was established. The methodology integrates optimal Latin hypercube sampling, finite element simulation, radial basis function surrogate modeling, and Sobol sensitivity analysis. This surrogate-assisted approach enables efficient exploration of high-dimensional parameter spaces while substantially reducing computational cost compared to direct finite element analysis.

Based on the prescribed curing cycle in Section 2.2 and engineering controllability considerations, six design variables were selected for quantitative evaluation, comprising three parameter types—holding temperature, heating rate, and holding time—each defined at two curing stages, yielding six design variables in total. The selected parameters and their ranges are summarized in Table 3.

To ensure comprehensive coverage of the high-dimensional design space under finite computational resources, the Optimal Latin Hypercube Sampling (LHS) method based on the Maximin distance criterion was employed to generate a representative set of input parameter samples, comprising N = 150 finite element simulations across k = 6 design parameters.

(4) $$X={x^{\left(i\right)}}_{i=1}^{N}x^{\left(i\right)}\in R^{k}$$

where (k) denotes the number of input parameters and (*N*) represents the total number of samples. During sampling, the sample distribution is optimized using the Maximin criterion, defined by the objective function:

(5) $$max\;(\underset{i\neq j}{\min}\left\|x_{i}-x_{j}\right\|)$$

Here, $x_{i}$ and $x_{j}$ represent the i-th and j-th sample points, respectively. This criterion effectively avoids local clustering of samples in the parameter space by maximizing the minimum Euclidean distance between sample points. After obtaining the parameter samples, a high-fidelity finite element heat transfer model is established for each set of sampled parameters to compute the corresponding temperature response. The mapping relationship can be expressed as:

(6) $$y^{\left(i\right)}=f\left(x^{\left(i\right)}\right),\;i=1,2,\ldots N$$

where f(·) denotes the nonlinear function describing the relationship between input parameters and temperature response, obtained from finite element simulations. Since Sobol global sensitivity analysis requires extensive model invocations, direct analysis based on the finite element model would incur prohibitively high computational costs. Therefore, this paper constructs a radial basis function (RBF) surrogate model based on the sample set(x,y), whose mathematical expression is:

(7) $$\hat{y}\left(x\right)=\sum_{i=1}^{N}w_{i}\varphi (\left\|x-x^{\left(i\right)}\right\|)$$

where $w_{i}$ represents the weighting coefficients. φ is the Gaussian kernel φ(r) = exp[−(c·r)^2^], evaluated after min–max normalizing the design space to [0,1]^6^, with all N = 150 finite element sample points retained as RBF centers, and ridge (Tikhonov) regularization applied when solving for the weights, ***w*** = (Φ + *λ***I**)^−1^*y*, where Φ is the N × N kernel matrix; The RBF surrogate model effectively approximates strongly nonlinear response relationships, significantly reducing computational time per model evaluation while maintaining prediction accuracy. This makes it suitable for large-scale parameter sampling and sensitivity analysis.

Upon completion of model construction, Sobol global sensitivity analysis was performed based on the trained RBF surrogate model to quantitatively assess the influence of individual input parameters and their interactions on temperature non-uniformity at the patch–adhesive interface. The analysis assumes that the model output can be expressed as a function of the multidimensional input parameters:

(8) $$Y=g(X_{1},X_{2},\ldots ,X_{k})$$

According to Sobol variance decomposition theory, the total variance of the output response can be decomposed into the sum of contributions from parameters of each order:

(9) $$Var\left(Y\right)=\sum_{i=1}^{k}V_{i}+\sum_{i<j}V_{ij}+\cdots +V_{1,2.\ldots ,k}$$

where $V_{i}$ denotes the first-order variance contribution of parameter $X_{i}$, and $V_{ij}$ represents the second-order interaction effect between parameters $X_{i}$ and $X_{j}$. Based on this, the first-order Sobol sensitivity index is defined to measure the direct impact of a single parameter on the output response:

(10) $$S_{i}=\frac{V_{i}}{\mathrm{Var}(Y)}$$

The total effect Sobol sensitivity index is defined as:

(11) $$S_{T_{i}}=1-\frac{\mathrm{Var}\left(Y|X_{\sim i}\right)}{\mathrm{Var}(Y)}$$

where $X_{\sim i}$ denotes all input parameters except $X_{i}$. The total effect index comprehensively reflects the main effect of parameter $X_{i}$ and its higher-order interaction effects with other parameters.

### 3.2. Multi-Objective Optimization

During composite patch repair, the high thermal conductivity of metallic support structures—such as the aluminum rib—induces a pronounced heat sink effect, resulting in non-uniform temperature distribution and significant thermal gradients at the patch–adhesive interface. To mitigate these effects and reduce the overall curing cycle, a multi-objective optimization model was formulated. The design variables comprise the two-stage holding temperatures (*T*_1_, *T*_2_), holding times (*dt*_1_, *dt*_2_), and heating rates (*r*_1_, *r*_2_). The objectives are to minimize both the maximum interfacial temperature difference (Δ*T*) and the total curing time (*t*_total_), subject to the constraint that the minimum degree of cure on the bottom surface of the patch at the patch–adhesive interface (DoC) reaches at least 0.98 to ensure full cure. This framework enables simultaneous improvement of thermal uniformity and process efficiency.

(12) $$\underset{x}{\min}\;F\left(x\right)=\left[∆T\left(x\right),t_{total}\left(x\right)\right]\;s.t.DoC\geq 0.98\;x={\left[dt_{1},dt_{2},T_{1},T_{2},r_{1},r_{2}\right]}^{T}$$

The three responses in Equation (12) are defined mathematically as follows. Δ*T* is the maximum, taken over the full cure-cycle duration, of the spatial range of temperature among all nodes on the patch–adhesive interface:

(13) $$\Delta T={max}_{t}[{max}_{i\in interface}\;T_{i}(t)-{min}_{i\in interface}\;T_{i}(t)]$$

DoC is the minimum degree of cure, evaluated at the end of the cure cycle, over all patch-resin elements/integration points within the interface region:

(14) $$DoC={min}_{i\in interface}\;\alpha_{i}(t_{end})$$

*t*_total_ is obtained in closed form directly from the six design variables through the prescribed five-stage schedule:

(15) $$t_{total}=\frac{\left(T_{1}-25\right)}{r_{1}}+dt_{1}+\frac{\left(T_{2}-T_{1}\right)}{r_{2}}+dt_{2}+\frac{\left(T_{2}-25\right)}{r_{cool}}$$

with *r*_cool_ = 1.0 °C/min held fixed, and all ramp and hold contributions expressed in consistent units.

**Figure 3:**
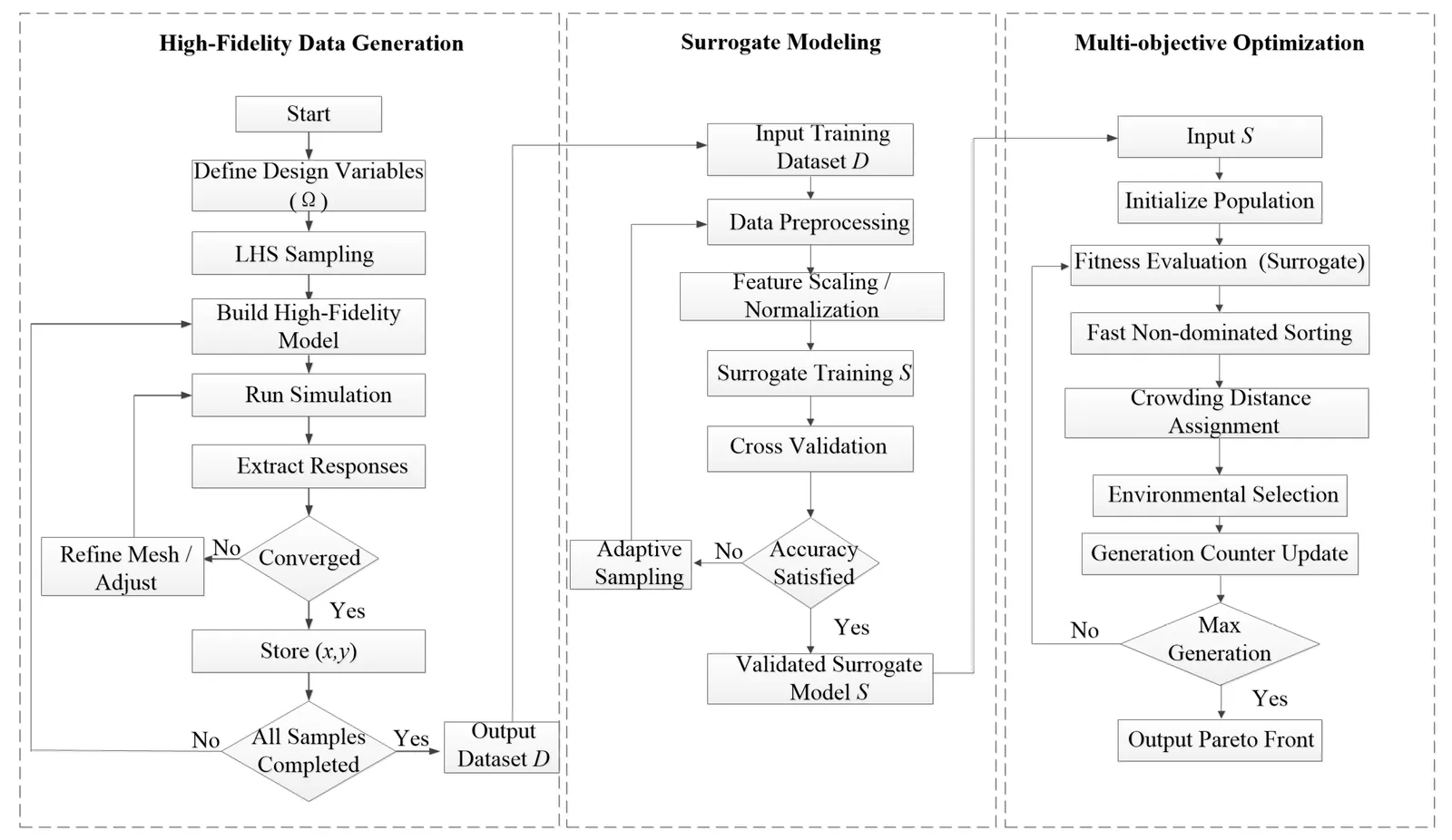
Multi-objective optimization workflow.

The multi-objective optimization workflow for the curing process is illustrated in Figure 3. First, optimal Latin hypercube sampling is employed to generate design points across the parameter space. These samples are subsequently used to construct Python 2.7 scripts that automate finite element simulations, from which key output responses (Δ*T*, *t*_total_, DoC) are extracted. A radial basis function (RBF) surrogate model is then established to approximate these input–output relationships, selected for its ability to handle high-order nonlinearity and multivariate interactions while balancing model complexity and accuracy. Finally, multi-objective optimization is performed using the NSGA-II algorithm, which employs non-dominated sorting to rank solutions based on Pareto dominance, enabling efficient identification of optimal trade-offs among competing objectives.

## 4. Results and Discussion

### 4.1. Finite Element Simulation Results

**Figure 4:**
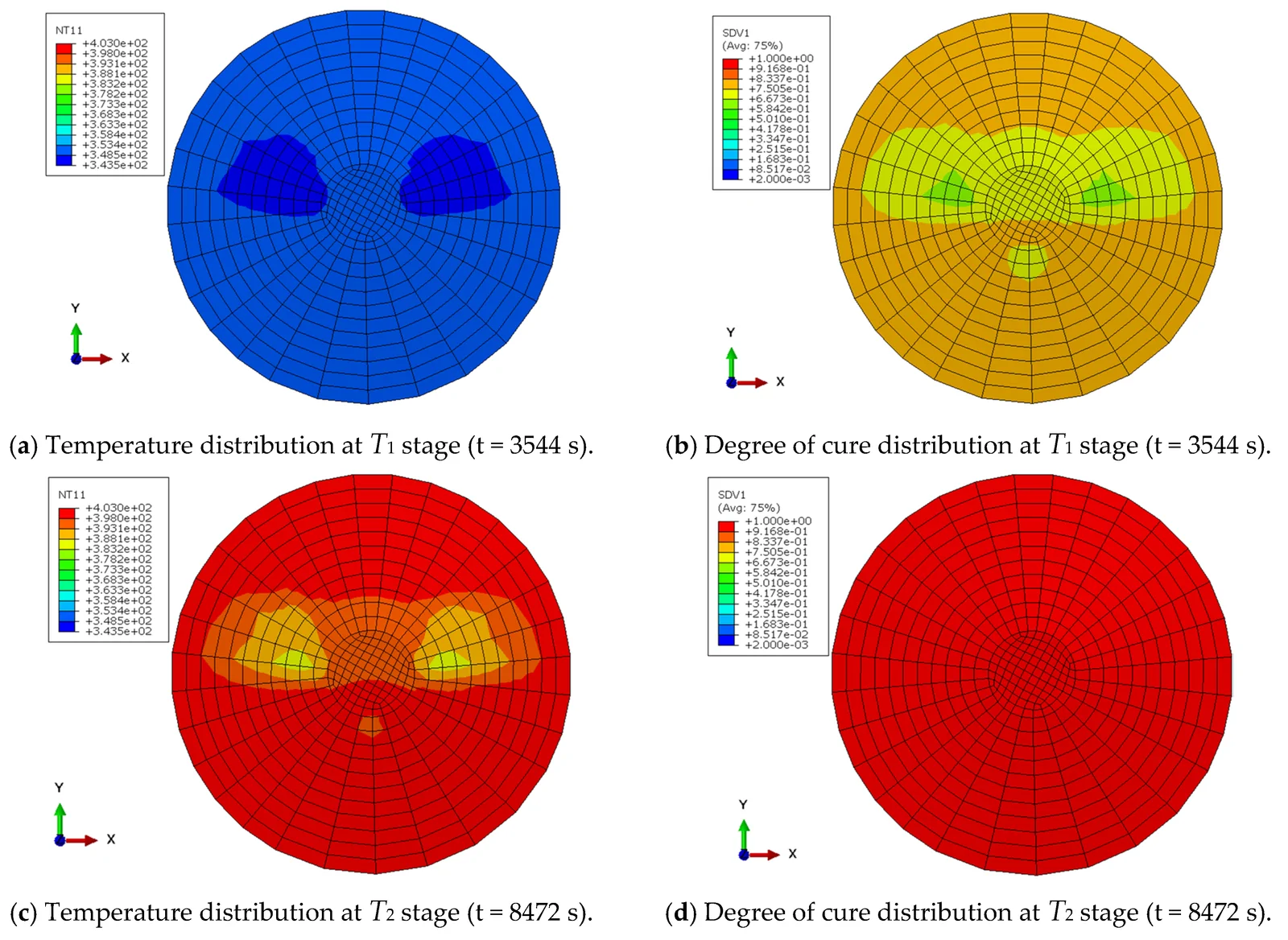
Temperature (NT11) and degree of cure (SDV1) distributions at the bottom surface of the patch during isothermal stages.

Figure 4 presents the temperature (NT11) and degree of cure (SDV1) distributions at the bottom surface of the patch during the two isothermal stages, *T*_1_ and *T*_2_. At the *T*_1_ stage (t = 3544 s), the interface temperature ranged from 343 to 353 K, with a maximum difference (Δ*T*) of approximately 10 K. During the *T*_2_ stage (t = 8472 s), the temperature increased to 383–403 K, and Δ*T* expanded to about 20 K, indicating that higher set temperatures amplify thermal gradients at the interface. Notably, symmetric low-temperature zones appeared on both sides of the circumference, spatially aligned with the underlying aluminum rib. This pattern arises from the high thermal conductivity of the metallic substructure, which induces a localized heat sink effect, accelerating heat dissipation, and consequently lowers the temperature at the adjacent patch–adhesive interface.

The corresponding curing degree distributions corroborate these thermal observations. At *T*_1_, DoC values ranged from 0.58 to 0.83, with lower degrees of cure concentrated in the same low-temperature zones, confirming that local thermal lag directly inhibits the curing reaction. By the *T*_2_ stage, despite the increased temperature differential, the elevated overall temperature promoted uniform cure across the interface, with DoC approaching full cure. This demonstrates that appropriately increasing the holding temperature and extending the holding time can effectively compensate for initial cure non-uniformity induced by the metallic heat sink, thereby ensuring complete interface consolidation.

### 4.2. Validation of the Finite Element Model

**Figure 5:**
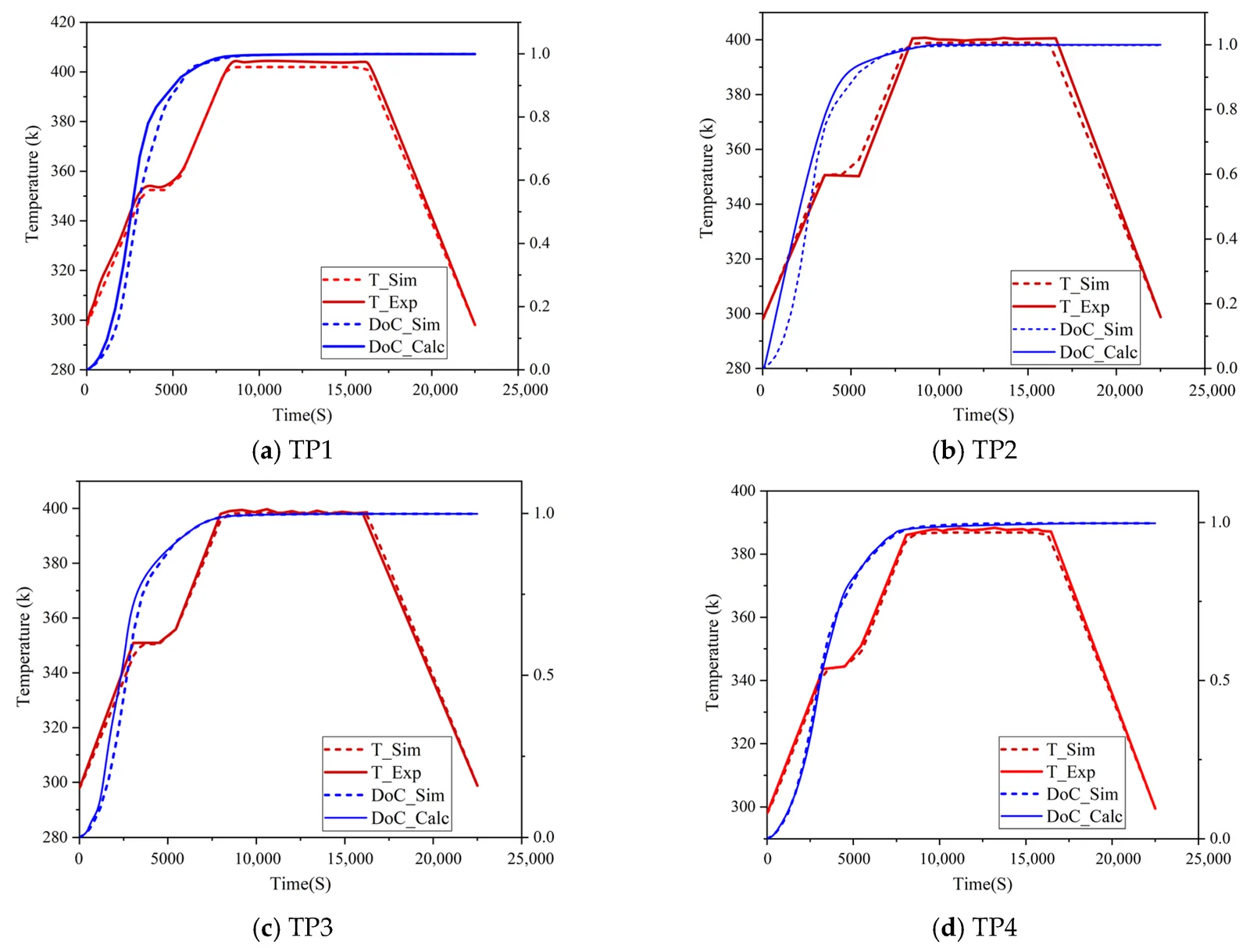
Comparison of simulated and calculated temperature and degree of cure histories at four patch–adhesive interface monitoring points.

To validate the reliability of the finite element model, four representative monitoring points—TP1, TP2, TP3, and TP4—were selected at the bottom surface of the patch. As noted in Section 2.3, this validation uses the original curing cycle only. The simulated temperature histories are compared with experimental measurements in Figure 5, with corresponding values summarized in Table 4. The “DoC_Calc” curves in Figure 5 were obtained by substituting the measured thermocouple temperature histories at each point into the same Kamal–Sourour kinetic model (Equations (2) and (3)) used in the FE simulation, integrated forward in time.

At the *T*_1_ dwell stage, the simulated temperatures at the four points were 352.7 K, 351.0 K, 350.1 K, and 343.5 K, respectively, compared to experimental values (mean ± standard deviation across three replicate specimens) of 353.7 ± 2.3 K, 350.5 ± 2.1 K, 350.7 ± 1.9 K, and 343.9 ± 1.7 K, yielding absolute errors of 1.0 K, 0.5 K, 0.6 K, and 0.4 K. During the *T*_2_ stage, simulated temperatures were 401.6 K, 398.8 K, 398.5 K, and 383.5 K, compared to experimental values of 403.0 ± 2.5 K, 400.1 ± 2.0 K, 399.1 ± 1.8 K, and 384.2 ± 1.5 K, yielding absolute errors of 1.4 K, 1.3 K, 0.6 K, and 0.7 K. Notably, at every measurement point and dwell stage, the absolute discrepancy between the simulated and mean experimental temperature is smaller than the standard deviation of the three replicate measurements at that point, indicating that the model prediction falls within the natural variability of repeated physical measurements rather than representing a systematic model bias. The degree-of-cure evolution at all four points showed consistent trends between the simulation results and the temperature-derived DoC (DoC_Calc), with full cure achieved in both cases. These results confirm that the developed finite element model accurately captures the thermal and curing behavior of the patch–adhesive interface, establishing its suitability for subsequent parameter sensitivity analysis and multi-objective optimization.

### 4.3. Parameter Sensitivity Results

**Figure 6:**
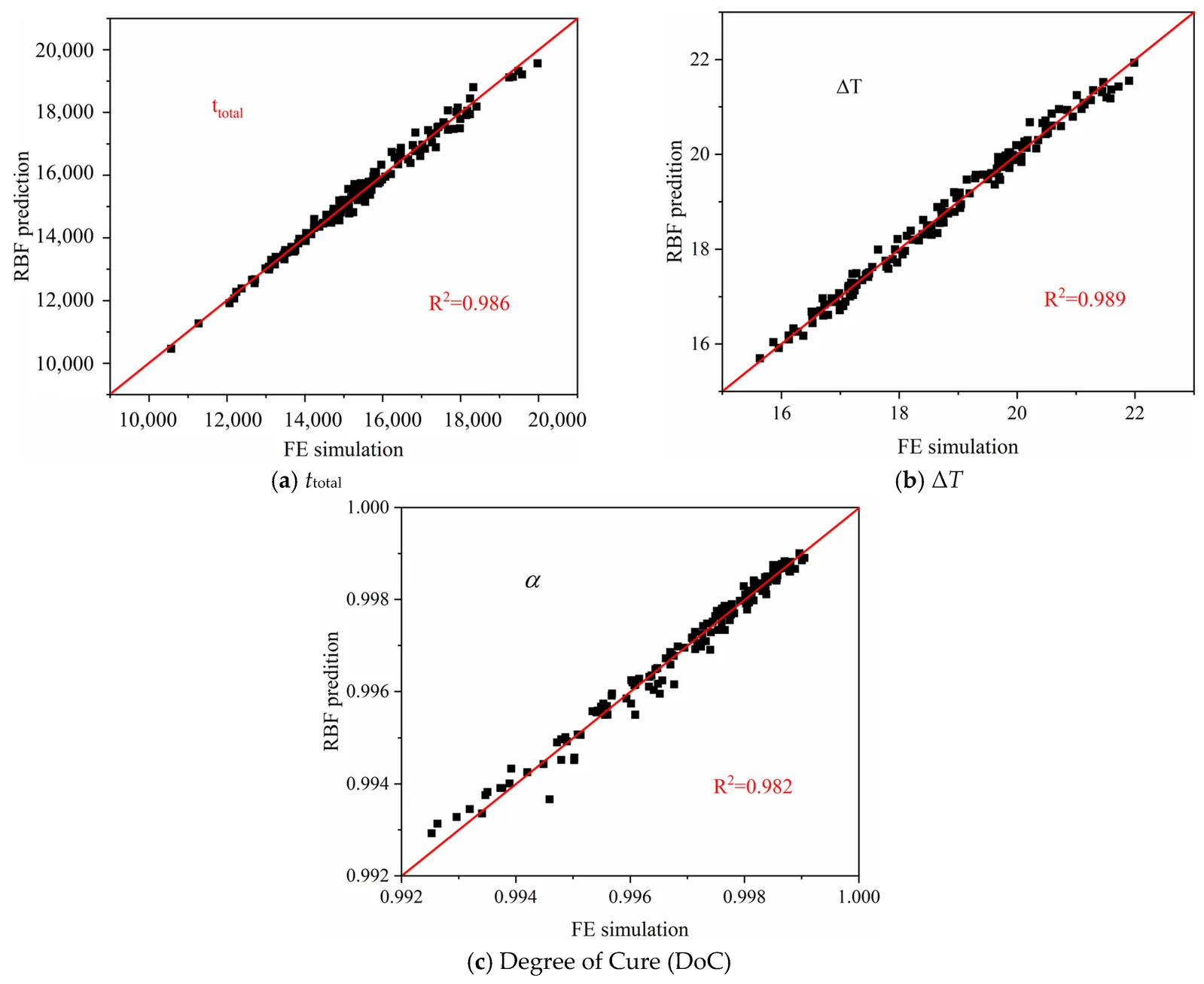
RBF prediction vs. finite element simulation of (**a**) total curing time, (**b**) maximum interfacial temperature difference, and (**c**) degree of cure.

To ensure the reliability of sensitivity analysis, a sufficient number of training samples is required. In this study, a radial basis function (RBF) surrogate model was constructed to replace the computationally expensive finite element simulations. Figure 6 presents scatter plots comparing the RBF-predicted values against the original finite element results for total curing time (*t*_total_), maximum interfacial temperature difference (Δ*T*), and degree of cure (DoC). The red diagonal line represents the ideal perfect-fit line, where closer clustering of data points indicates higher predictive accuracy.

The coefficient of determination (R^2^) was employed to quantitatively evaluate surrogate model fidelity. As shown in Figure 6, the RBF model exhibits excellent predictive capability, with all scatter points tightly clustered around the ideal line and R^2^ values exceeding 0.98 for all three output responses. These results confirm that the RBF surrogate model accurately reproduces the finite element simulations and can be reliably used for subsequent global sensitivity analysis to identify the key parameters governing the curing process.

To ensure that the reported surrogate accuracy reflects genuine predictive capability rather than mere interpolation of the training data, the RBF hyperparameters (shape parameter c and regularization parameter λ) for each response (*t*_total_, ΔT, and DoC) were selected via 5-fold cross-validation, minimizing the mean held-out RMSE across folds, rather than by matching a pre-specified in-sample R^2^ target. The cross-validation-selected values are c = 0.5, λ = 4.33 × 10^−5^ for *t*_total_; c = 0.5, λ = 1.87 × 10^−3^ for ΔT; and c = 0.1, λ = 1.00 × 10^−5^ for DoC. Final model coefficients were then estimated using the complete set of 150 FE simulations at the cross-validation-selected hyperparameters. Table 5 reports both the in-sample goodness-of-fit (computed after refitting on all 150 samples) and the 5-fold cross-validation accuracy (computed on held-out folds) for each response. The small gap between in-sample and cross-validated R^2^ (Δ ≤ 0.017 for all three responses) indicates that the surrogate models generalize well to unseen design points.

**Figure 7:**
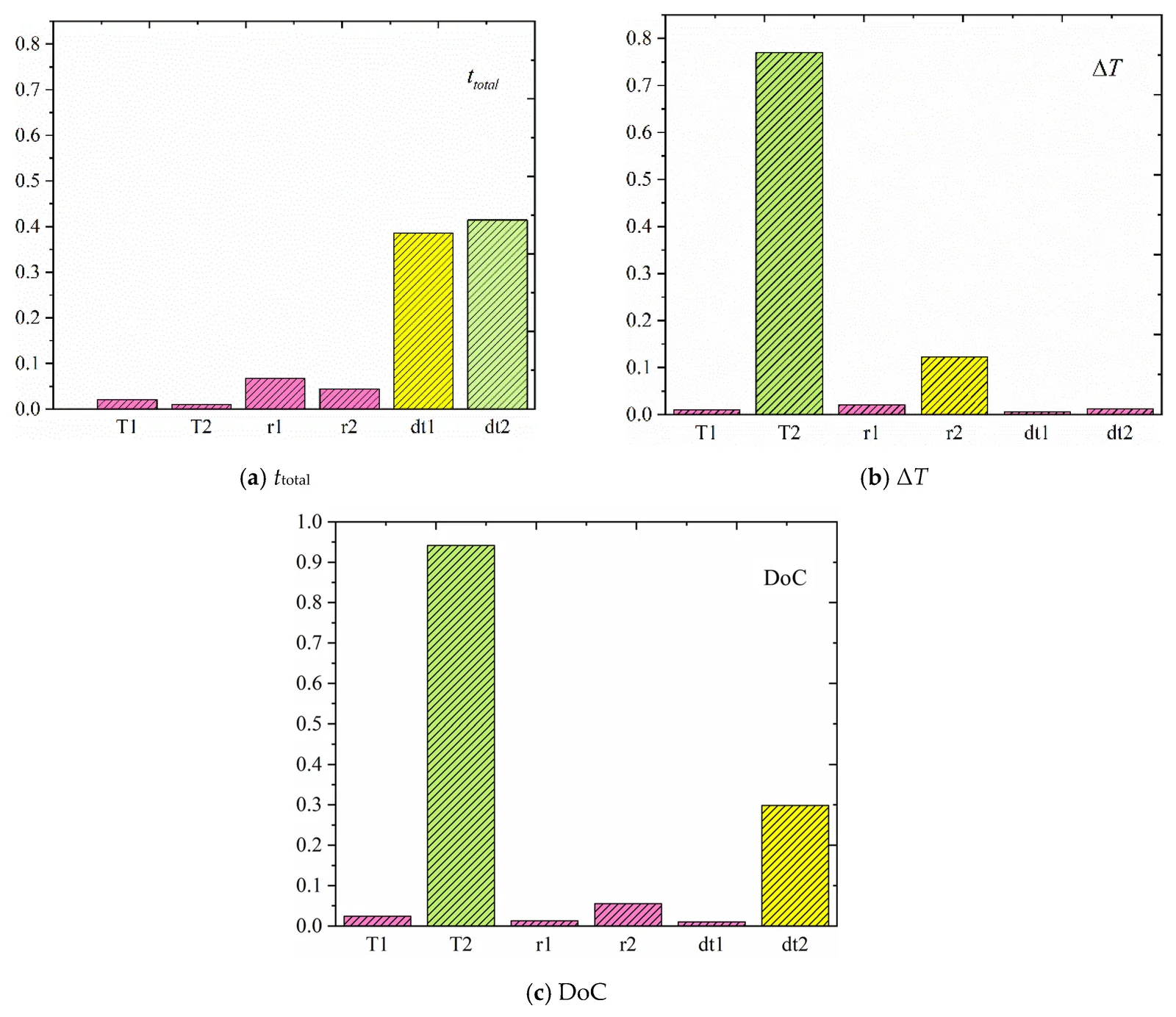
First-order Sobol sensitivity indices (S_1_) for (**a**) *t*_total_; (**b**) Δ*T*; (**c**) DoC.

Figure 7 presents the global sensitivity analysis results for total curing time (*t*_total_), maximum interfacial temperature difference (Δ*T*), and degree of cure (DoC). The three output responses exhibit distinct dependencies on the process parameters. Δ*T* is predominantly governed by the second-stage holding temperature (*T*_2_), with sensitivity far exceeding that of other parameters. The heating rate (*r*_2_) also contributes, indicating that peak temperature conditions and the heating profile are the primary determinants of interfacial thermal uniformity. *t*_total_ is most sensitive to the holding times (*dt*_1_ and *dt*_2_), with secondary contributions from the heating rates (*r*_1_ and *r*_2_). This reflects the dominant role of time-related parameters in dictating overall process duration, while temperature levels exert comparatively limited influence. DoC exhibits a strong monotonic dependence on *T*_2_; dt2 also contributes to the variance in DoC, but its effect remains small relative to that of *T*_2_. This confirms that the final cure state is primarily regulated by the critical temperature threshold.

The near-zero sensitivity of *T*_1_ to Δ*T* can be explained by the transient nature of the heat sink effect. During the first holding stage (80 °C), the temperature difference between the interface and the surrounding metallic substructure remains small, so the heat sink-induced gradient has not yet fully developed; the governing thermal driving force at this stage is dominated by the initial heating rate rather than the absolute holding temperature. Only once the interface temperature approaches the second dwell (130 °C) does the conductivity mismatch between the aluminum rib and the composite produce a pronounced, geometry-locked gradient. Consequently, Δ*T* is governed almost entirely by the second-stage parameters (*T*_2_, *r*_2_), while the first stage primarily influences the initial degree of cure rather than the final interfacial temperature non-uniformity. This follows from the approximately 180- to 230-fold conductivity contrast between the aluminum rib and the composite’s through-thickness direction (Table 1 and Table 2): the rib dissipates heat almost isothermally while the composite resists through-thickness flow, so the heat-sink effect strengthens with temperature; consequently, *T*_2_ and *r*_2_, rather than *T*_1_, dominate Δ*T*.

In summary, *T*_2_ emerges as the core factor governing both interfacial thermal behavior and curing quality, whereas time-related parameters play a supplementary but essential role in controlling the curing kinetics.

**Figure 8:**
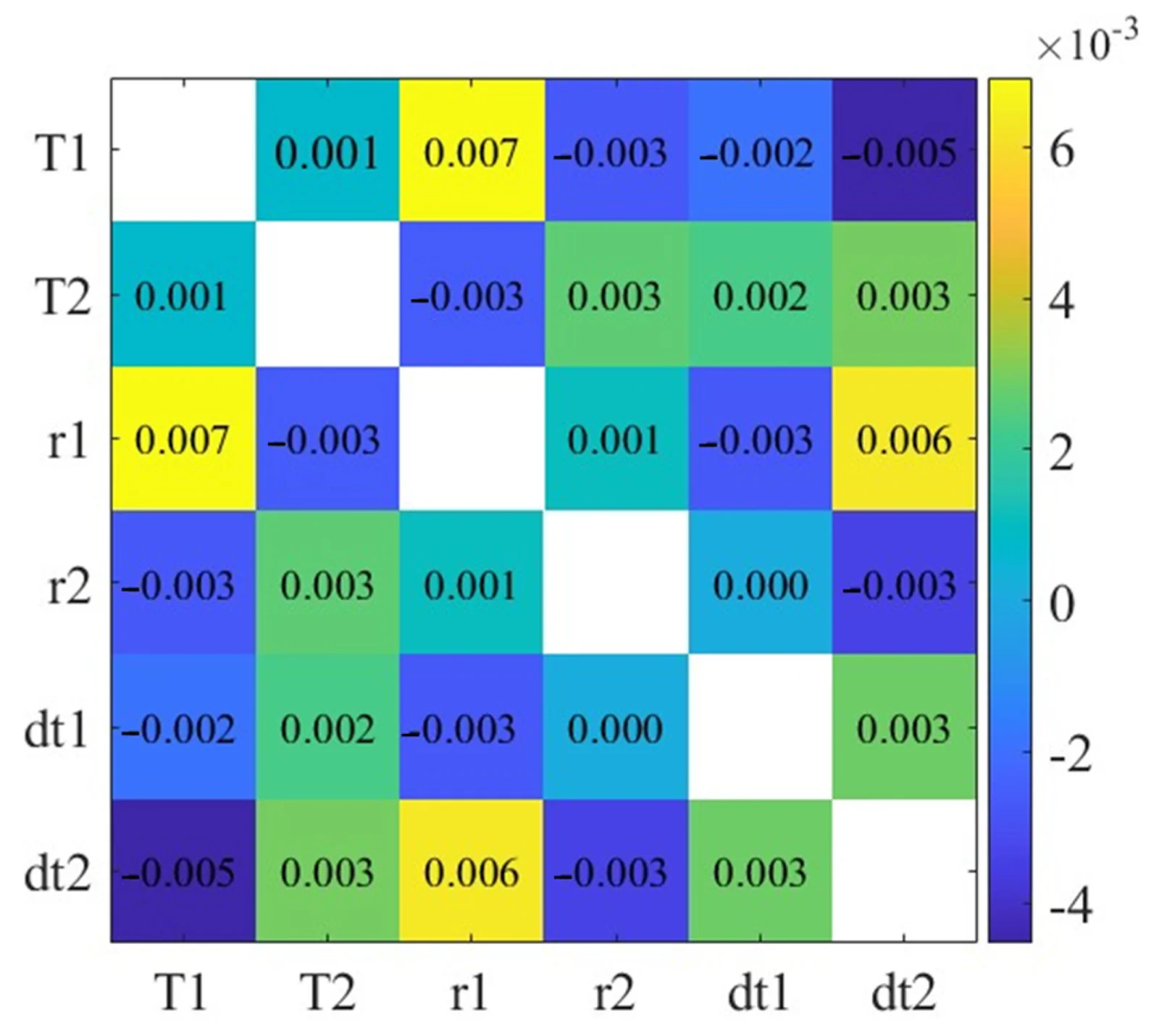
Second-order Sobol interaction indices for all parameter pairs governing Δ*T*.

To further examine whether the dominant parameters *T*_2_ and *r*_2_ act independently or synergistically, second-order Sobol interaction indices were computed for all fifteen parameter pairs (Figure 8). For T_2_, the total-effect index (S_T_ = 0.806) exceeds its first-order index (S_1_ = 0.790) by only 0.017, and for r_2_ the two indices are essentially equal (S_1_ = 0.204, S_T_ = 0.199); Although the point estimate of S_1_ for r_2_ is slightly higher than S_T_, bootstrap analysis (1000 paired Saltelli/Jansen resamples) gives 95% CIs of [0.098, 0.307] and [0.193, 0.201], respectively, indicating that the apparent S_1_ > S_T_ is due to sampling error rather than a violation of the theoretical S_1_ ≤ S_T_ relationship. For both *T*_2_ and *r*_2_, the estimated total-effect and first-order indices are very close, and the paired-bootstrap analysis indicates that the apparent negative gap for *r*_2_ is attributable to sampling uncertainty. This is confirmed directly by the pairwise interaction map in Figure 8, where all second-order indices—including the *T*_2_ × *r*_2_ pair (0.0027)—remain within ±0.007, an order of magnitude smaller than the first-order effects of *T*_2_ and r_2_ themselves. The largest interaction magnitudes observed, *r*_1_ × *dt*_2_ (0.0064) and *T*_1_ × *r*_1_ (0.0069), are associated exclusively with parameters that individually exert negligible main effects on Δ*T* (|S_1_| < 0.003 for *T*_1_, *r*_1_, *dt*_1_, *dt*_2_), and are consistent with the sampling noise floor expected from Monte Carlo-based Sobol estimation at this sample size, as evidenced by the slightly negative first-order indices reported for these same parameters (e.g., *T*_1_:S_1_ = −0.0026). These results demonstrate that ΔT is governed by an essentially additive combination of T_2_ and r_2_, without meaningful synergistic or antagonistic coupling between the two—a finding that simplifies the practical interpretation of the sensitivity analysis, as the two dominant parameters can be reasoned about and adjusted largely independently when tuning the curing profile for thermal uniformity.

### 4.4. Multi-Objective Optimization Results

Multi-objective optimization was performed using the NSGA-II algorithm (via MATLAB’s gamultiobj solver), with the following hyperparameters: population size = 100, maximum generations = 200, crossover fraction = 0.8, Pareto fraction = 0.35, and function tolerance = 1 × 10^−6^. The degree-of-cure constraint (DoC ≥ 0.98) was enforced as a nonlinear inequality constraint throughout the search, and all solutions on the final Pareto front satisfied this constraint (minimum DoC across the returned Pareto set exceeded 0.98).

**Figure 9:**
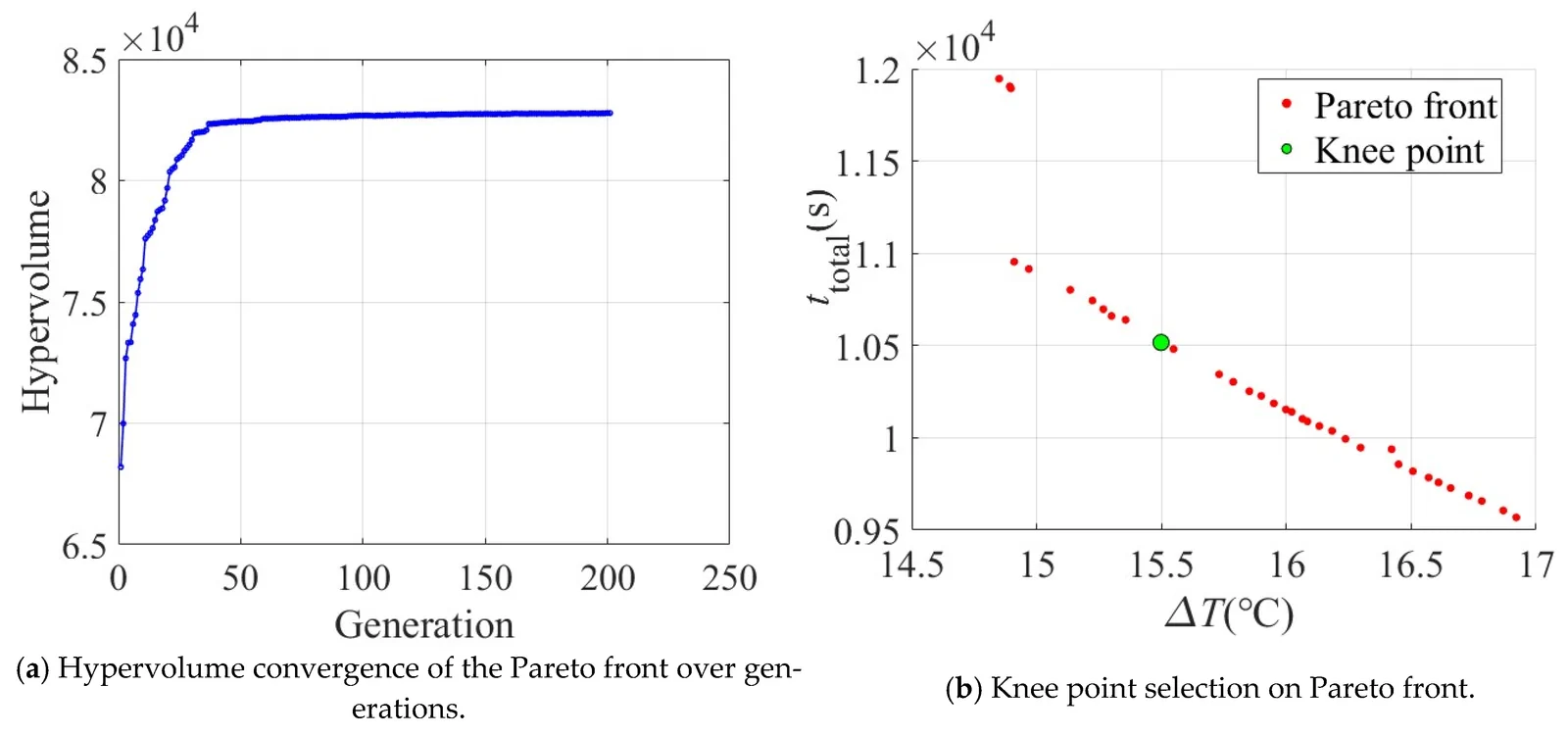
Multi-objective optimization results.

Figure 9a shows the hypervolume of the population’s Pareto front as a function of generation, using a fixed reference point across all generations. The hypervolume increases rapidly over the first ~40 generations and plateaus thereafter, indicating that the population had effectively converged well before the algorithm reached its maximum generation limit of 200.

Figure 9b shows the resulting Pareto front between ΔT and *t*_total_, comprising 35 non-dominated solutions. A single compromise solution (the knee point, highlighted in green) was selected as the point of minimum Euclidean distance to the utopia point in normalized objective space, representing the design at which further improvement in one objective would come at disproportionate cost to the other. The knee-point design variables are shown in Table 6.

Table 6 summarizes the process parameters, thermal uniformity, and cure quality of the original and NSGA-II-optimized curing cycles. The original cycle, following a conventional two-dwell profile (80 °C/130 °C, 1.0 °C/min heating rate), achieves complete cure (DoC = 1.00) but requires 22,500 s and produces a maximum interfacial temperature difference of 20.0 °C. The surrogate-predicted optimized cycle reduces Δ*T* by 4.50 °C (22.5%) and total curing time by 8091 s (36%), while the patch–adhesive interface DoC decreases only marginally, from 1.00 to 0.9925—still comfortably above the 0.98 threshold enforced as a constraint throughout the NSGA-II search.

Examining the individual parameters clarifies how this trade-off is achieved. Rather than preserving the original two-dwell structure, the optimized cycle compresses the first holding stage to a negligible duration (*dt*_1_ = 6 s, versus 1800 s originally), raises both heating rates (*r*_1_: 1.00 → 3 °C/min; *r*_2_: 1.00 → 1.50 °C/min), and lowers both holding temperatures (*T*_1_: 80.0 → 70.0 °C; *T*_2_: 130.0 → 115 °C), while extending the final hold (*dt*_2_ = 6303 s). In effect, the optimization replaces the traditional low-temperature dwell—originally intended to moderate exothermic-driven thermal gradients before the high-temperature hold—with a rapid, near-continuous ramp directly into an extended, lower-temperature final hold. This indicates that, for the present material system and repair geometry, thermal uniformity is governed more strongly by the peak holding temperature and second-stage heating rate than by the presence of a distinct low-temperature dwell, consistent with the sensitivity analysis in Section 4.3 identifying *T*_2_ and *r*_2_ as the dominant parameters.

Given the response characteristics of an industrial hot-bonder controller—thermal inertia of the blanket and laminate, sensor/controller lag, and the risk of overshoot—a *dt*_1_ of just 6 s is practically indistinguishable from no first dwell at all. We therefore recommend that, in implementation, the first holding stage be omitted entirely, with the heating profile programmed as a single continuous ramp from ramp 1 (25 °C → *T*_1_) directly into ramp 2 (*T*_1_ → *T*_2_), rather than commanding a literal 6 s isothermal hold that is unlikely to be reliably achievable in practice.

### 4.5. Robustness to the Random Seed

Because NSGA-II is a stochastic algorithm, we assessed the sensitivity of the Pareto front and knee-point solution to the random seed by repeating the optimization in MATLAB R2022b for five independent seeds. Table 7 shows multi-seed robustness check of the NSGA-II knee-point solution 5 independent random seeds. Despite this variation in individual design variables, the resulting knee-point performance was highly consistent across the five runs: Δ*T* = 15.5 ± 0.13, DoC = 0.9923 ± 0.00005, with all five solutions comfortably satisfying the DoC ≥ 0.98. The total curing time was 14,358 ± 96, notably more stable than the individual *T*_1_, *r*_2_, and *dt*_1_ values whose combined contribution determines the total curing time. The Pareto-front hypervolume had a coefficient of variation of 5.97% across the five seeds.

**Figure 10:**
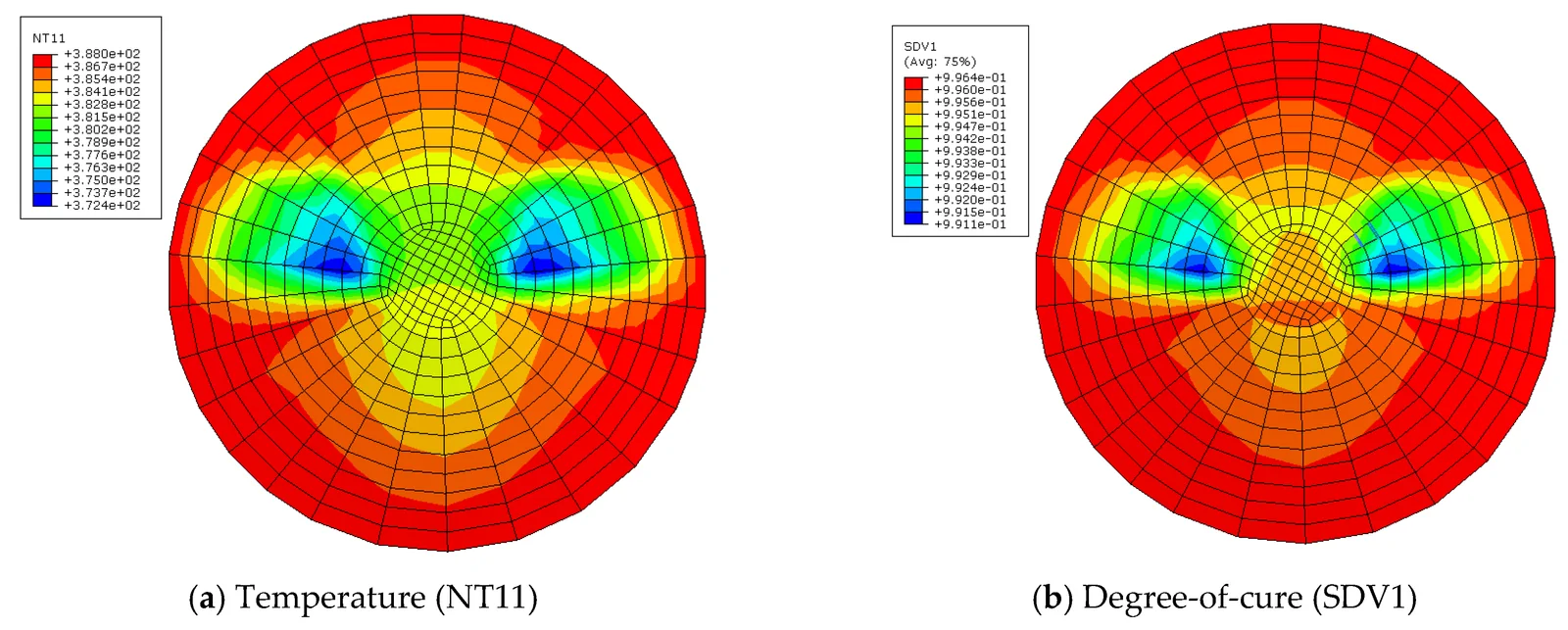
High-fidelity FE-verified (**a**) temperature (NT11) and (**b**) degree-of-cure (SDV1) distributions at the patch–adhesive interface for the knee-point optimized cycle (T2 dwell stage).

To confirm that the knee-point improvements are not artifacts of surrogate approximation, the design variables in Table 6 were re-evaluated directly with the high-fidelity FE model. Figure 10 shows the resulting patch–adhesive interface temperature (NT11) and degree-of-cure (SDV1) distributions at the T2 dwell stage, reproducing the same twin low-temperature and low-DoC lobes adjacent to the aluminum rib seen in Figure 4. The FE model yields ΔT = 15.6 °C and DoC = 0.991, within 0.6% and 0.15% of the surrogate predictions (15.5 °C and 0.9925, respectively) and comfortably above the DoC ≥ 0.98 constraint, confirming the reliability of the surrogate-based optimization.

Taken together, the multi-seed robustness analysis and the subsequent high-fidelity FE verification demonstrate that the proposed NSGA-II framework consistently identifies high-performing curing profiles that substantially improve thermal uniformity and process efficiency relative to the conventional cycle while satisfying the prescribed cure-quality constraint.

### 4.6. Limitations

This study has several limitations. (1) Cure-induced residual stresses and resin viscoelastic evolution were not modeled, and cooling rate was held fixed; coupling with a residual-stress/viscoelastic framework is left for future work. (2) The FE model assumes perfect interfacial heat-flux continuity and neglects thermal contact resistance, so reported Δ*T* values represent best-case estimates. (3) The optimized curing profile (Table 6) was verified only numerically (Section 4.5) and has not been experimentally realized. (4) The analysis covers one scarf geometry, one aluminum-rib substructure, and one material combination; the dominant role of *T*_2_ and *r*_2_ has not been demonstrated for other rib thicknesses, patch sizes, or substrate materials.

## 5. Conclusions

This study developed and experimentally validated a coupled heat transfer–curing kinetics finite element model to characterize the metal-induced heat sink effect at the bottom surface of the patch during CFRP scarf repair, and established a surrogate-assisted NSGA-II framework to optimize the curing profile accordingly. For the present scarf-repair configuration, sensitivity analysis identified the second-stage holding temperature (*T*_2_) and heating rate (*r*_2_) as the dominant parameters governing interfacial thermal uniformity, with negligible interaction effects between them, while the holding times (*dt*_1_, *dt*_2_) primarily control total curing time. The optimized curing profile reduces the maximum interfacial temperature difference by 22.5% and total curing time by 36%, while maintaining the degree of cure above the specified 0.98 threshold—demonstrating that substantial gains in thermal uniformity and process efficiency can be achieved without compromising cure quality.

## Figures and Tables

**Table 1:** Thermophysical properties of adhesive film and aluminum alloy.

Thermal Physical Properties for LWF-2B Adhesive Film [31]		Thermal Physical Properties for Al
Parameter	Value	Value
*ρ_r_* [kg/m^3^]	1200	2700
*C* [J/(kg·K)]	1877	900
*k_yy_* [W/(m·K)]	0.35	150

**Table 2:** Thermophysical and curing kinetic properties of composite and patch materials.

Thermal Physical Properties for ZT7H/5429 Composite [32]		Thermal Physical Properties for ZT7H/ACTECH 1206 Composite [33]	Cure Kinetic Constants for ACTECH 1206 Resin [34]	
Parameter	Value	Value	Parameter	Value
*ρ*_c_ [kg/m^3^]	1578	1560	*A*_1_ [s^−1^]	3.2 × 10^6^
*C* [J/(kg·K)]	1120	889	*E*_1_ [J/mol]	63,000
*k*_xx_ [W/(m·K)]	6.2	6.38	*n*	1.85
*k*_yy_ [W/(m·K)]	0.83	0.65	*H*_R_ [J/kg]	226,000
*k*_zz_ [W/(m·K)]	0.83	0.65	*R* [J/(mol·K)]	8.314
*ρ*_r_ [kg/m^3^]		1230	*A*_2_ [s^−1^]	1.5 × 10^8^
*V*_f_		58%	*E*_2_ [J/mol]	78,000
Initial degree of cure		0.01	*m*	0.92

**Table 3:** Design of process parameters for optimization.

Variable	Symbol	Range	Symbol	Range
Holding Temperature	*T*_1_	333–373 K	*T*_2_	378–428 K
Heating Rate	*r*_1_	1–5 °C/min	*r*_2_	1–5 °C/min
Hold time	*dt*_1_	0–1800 s	*dt*_2_	6300–9900 s

**Table 4:** Measured and predicted temperatures at four monitoring points.

Measured Point	*T*_1_ Simulated (K)	*T*_1_ Experimental (K)	*T*_1_ Absolute Error (K)	*T*_2_ Simulated (K)	*T*_2_ Experimental (K)	*T*_2_ Absolute Error (K)
1	352.7	353.7 ± 2.3	1.0	401.6	403.0 ± 2.5	1.4
2	351.0	350.5 ± 2.1	0.5	398.8	400.1 ± 2.0	1.3
3	350.1	350.7 ± 1.9	0.6	398.5	399.1 ± 1.8	0.6
4	343.5	343.9 ± 1.7	0.4	383.5	384.2 ± 1.5	0.7

**Table 5:** Surrogate model accuracy: in-sample fit vs. 5-fold cross-validation.

Response	In-Sample R^2^	In-Sample RMSE	CV R^2^	CV RMSE	Generalization Gap (R^2^)
*t*_total_ (s)	1.0000	12.13	0.9983	65.79	0.0017
Δ*T* (K)	0.9934	0.1285	0.9766	0.2365	0.0168
DoC	0.9822	0.0002	0.9670	0.0420	0.0152

**Table 6:** Original vs. optimized process parameters.

Symbol (Unit)	*T*_1_ (K)	*T*_2_ (K)	*r*_1_ (°C/min)	*r*_2_ (°C/min)	*dt*_1_ (s)	*dt*_2_ (s)	Δ*T* (K)	*t*_total_ (s)	DoC
Original cycle	353	403	1	1	1800	8100	20	22,500	1
optimized cycle	343	388	3	1.5	6	6303	15.5	14,409	0.9925

**Table 7:** Multi-seed robustness check of the NSGA-II knee-point solution (5 independent random seeds).

Symbol (Unit)	*T*_1_ (K)	*T*_2_ (K)	*r*_1_ (°C/min)	*r*_2_ (°C/min)	*dt*_1_ (s)	*dt*_2_ (s)	Δ*T* (K)	DoC	*t*_total_	Pareto-Front Hypervolume
Mean ± SD	359.99 ± 3.57	388.03 ± 0.04	2.996 ± 0.003	1.713 ± 0.056	26.9 ± 26.2	6319.3 ± 12.6	15.5 ± 0.13	0.9923 ± 0.00005	14,358 ± 96	5954 ± 356
CV	0.99%	0.01%	0.08%	3.25%	97.4%	0.20%	0.85%	0.01%	0.67%	5.97%

## Data Availability

The original contributions presented in this study are included in the article. Further inquiries can be directed to the corresponding author.

## Abbreviations

CFRP	Carbon fibre-reinforced polymer
LHS	Latin Hypercube Sampling
RBF	Radial basis function
